# Lung cancer and the Gut-microbiota-lung Axis: emerging evidence and potential clinical implications

**DOI:** 10.3389/fmed.2025.1655780

**Published:** 2025-11-24

**Authors:** Li Liu, Li Yang, Hongdu Zhang, Hongmin Li, Tianlu Shang, Lihan Liu

**Affiliations:** 1 Department of thoracic surgery, The Third Affiliated Hospital of Gansu University of Chinese Medicine, Baiyin, China; 2 Department of Oncology, The Third Affiliated Hospital of Gansu University of Chinese Medicine, Baiyin, China

**Keywords:** lung cancer, Gut-microbiota-lung Axis, gut microbiota, immunotherapy, short-chain fatty acids, gut dysbiosis

## Abstract

Lung cancer remains the leading cause of cancer-related deaths globally, with a 5-years survival rate of only around 20%. Merging cohort and Mendelian-randomization studies indicate that gut dysbiosis is associated with—though not yet proven to cause—an elevated risk and worse prognosis of non-small-cell lung cancer. Lower fecal abundance of butyrate producers such as *Faecalibacterium prausnitzii* and expansion of Enterobacteriaceae correlate with reduced systemic CD8 + T-cell infiltration and shorter progression-free survival during immune-checkpoint blockade. Antibiotic exposure within 30 days before anti-PD-1 initiation is consistently linked to diminished objective response and overall survival in retrospective cohorts, whereas supplementation with butyrogenic probiotics or fecal microbiota transplantation from responders restores therapeutic efficacy in pre-clinical models. This review integrates epidemiological, mechanistic and clinical data to clarify the current evidence, identify gaps and outline the steps needed to translate gut–lung-axis research into safe, effective adjunctive therapies for patients with lung cancer.

## 1 Introduction

Lung cancer remains the leading cause of cancer-related deaths globally, with an estimated 1.8 million deaths annually. Non-small-cell lung cancer (NSCLC) accounts for over 85% of cases (1). While recent years have witnessed significant advancements in lung cancer treatment, such as the emergence of targeted therapies and immune checkpoint inhibitors, the prognosis for lung cancer patients remains poor, with a global 5-year overall survival rate of 19.8% (95% CI 19.6–20.0) for all stages combined, ranging from 4.2% (stage IV) to 68.4% (stage I) in the most recent CONCORD-3 analysis covering 2000–2014 diagnoses. Regional figures for China (2012–2015) mirror the global estimate at 19.7% overall (2). For example, the CheckMate-816 trial showed that neoadjuvant nivolumab plus chemotherapy increased pathological complete response rates, yet the absolute survival gain was modest (3). Thus, there is an urgent need to explore novel therapeutic strategies to enhance treatment efficacy and improve patient survival.

The gut–lung axis denotes bidirectional communication between intestinal microbiota and pulmonary immunity (4). Cross-sectional studies report that fecal depletion of butyrate producers such as *Faecalibacterium prausnitzii* or enrichment of *Fusobacterium spp.* is associated with NSCLC (5, 6). Similarly, Mendelian-randomization analyses indicate that genetically predicted lower abundance of *Bacteroides* and *Faecalibacterium* is associated with higher NSCLC risk, mediated by reduced CD8 + T-cell infiltration (7, 8). Whether these associations reflect causality or reverse causation is unresolved; nevertheless, germ-free mice exhibit impaired pulmonary immunity and accelerated urethane-driven adenocarcinoma (4). Furthermore, recent advances in microbiome research have provided new insights into the relationship between the gut microbiota and lung cancer (9). Studies have shown that the gut microbiota composition in lung cancer patients differs significantly from that in healthy individuals. For example, some research has found that the relative abundance of certain bacterial genera, such as *Fusobacterium* and *Porphyromonas*, is higher in lung cancer patients (5, 6). Moreover, the gut microbiota can influence the efficacy of lung cancer treatment. A study demonstrated that patients with a specific gut microbiota profile had better responses to immune checkpoint inhibitors (ICIs) and longer progression-free survival (PFS) (10). Additionally, gut microbiota metabolites, such as short-chain fatty acids (SCFAs) and bile acids, can affect lung cancer progression by regulating immune responses and inflammation (11). Collectively, current evidence supports an association rather than a proven causal role of gut dysbiosis in lung-cancer initiation or progression.

The Gut-microbiota-lung Axis holds great promise for the treatment of lung cancer (12). Gut microbiota modulation through probiotics, prebiotics, and fecal microbiota transplantation (FMT) has shown potential in regulating immune responses and improving treatment efficacy in lung cancer patients. For example, a study found that supplementation with specific probiotics could enhance the efficacy of immune checkpoint inhibitors (12). Furthermore, understanding the Gut-microbiota-lung Axis may help identify novel biomarkers for lung cancer diagnosis and prognosis. However, there are still some challenges in this field (13). The mechanisms underlying the Gut-microbiota-lung Axis in lung cancer are complex and require further exploration. Additionally, the safety and long-term efficacy of gut microbiota interventions need to be validated through large-scale clinical trials.

In this review, we aim to comprehensively evaluate the current evidence on the Gut-microbiota-lung Axis in lung cancer, explore its potential clinical implications, and identify future research directions. We will discuss the role of the gut microbiota in lung cancer development and progression, its impact on treatment efficacy, and the potential mechanisms involved. We will also examine the clinical applications of gut microbiota modulation in lung cancer and the challenges and opportunities in this field. By bridging basic science and clinical applications, we hope to provide new perspectives for the prevention, diagnosis, and treatment of lung cancer.

## 2 Transparent evidence synthesis

This review is based on a structured literature search of PubMed (up to 31 March 2025) using the strategy: (lung cancer OR non-small cell lung cancer) AND (gut microbiota OR gut-lung axis OR fecal microbiota) AND (immunotherapy OR chemotherapy OR prognosis). Inclusion criteria: peer-reviewed English-language articles (2010–2025) reporting original human or pre-clinical data on gut microbiota composition, metabolites or interventions in lung cancer. Exclusion criteria: conference abstracts, reviews without primary data, studies lacking lung-cancer-specific outcomes. Because the field is composed predominantly of observational and single-arm trials, the risk of publication bias toward positive associations is acknowledged. Heterogeneity is evident in sequencing platforms (16S rRNA V3-V4 vs. shotgun metagenomics), DNA extraction protocols, bioinformatic pipelines (QIIME 2 vs. MOTHUR), and metabolomic platforms (GC-MS vs. LC-MS/MS), precluding formal meta-analysis. These limitations are reflected in the use of qualitative synthesis throughout the manuscript. Prior reviews have summarized cross-sectional associations between gut dysbiosis and lung cancer risk (14), the present work extends those observations by integrating longitudinal intervention data and by explicitly distinguishing prognostic from predictive microbial signatures.

## 3 The Gut-microbiota-lung Axis: physiological and immunological foundations

**FIGURE 1:**
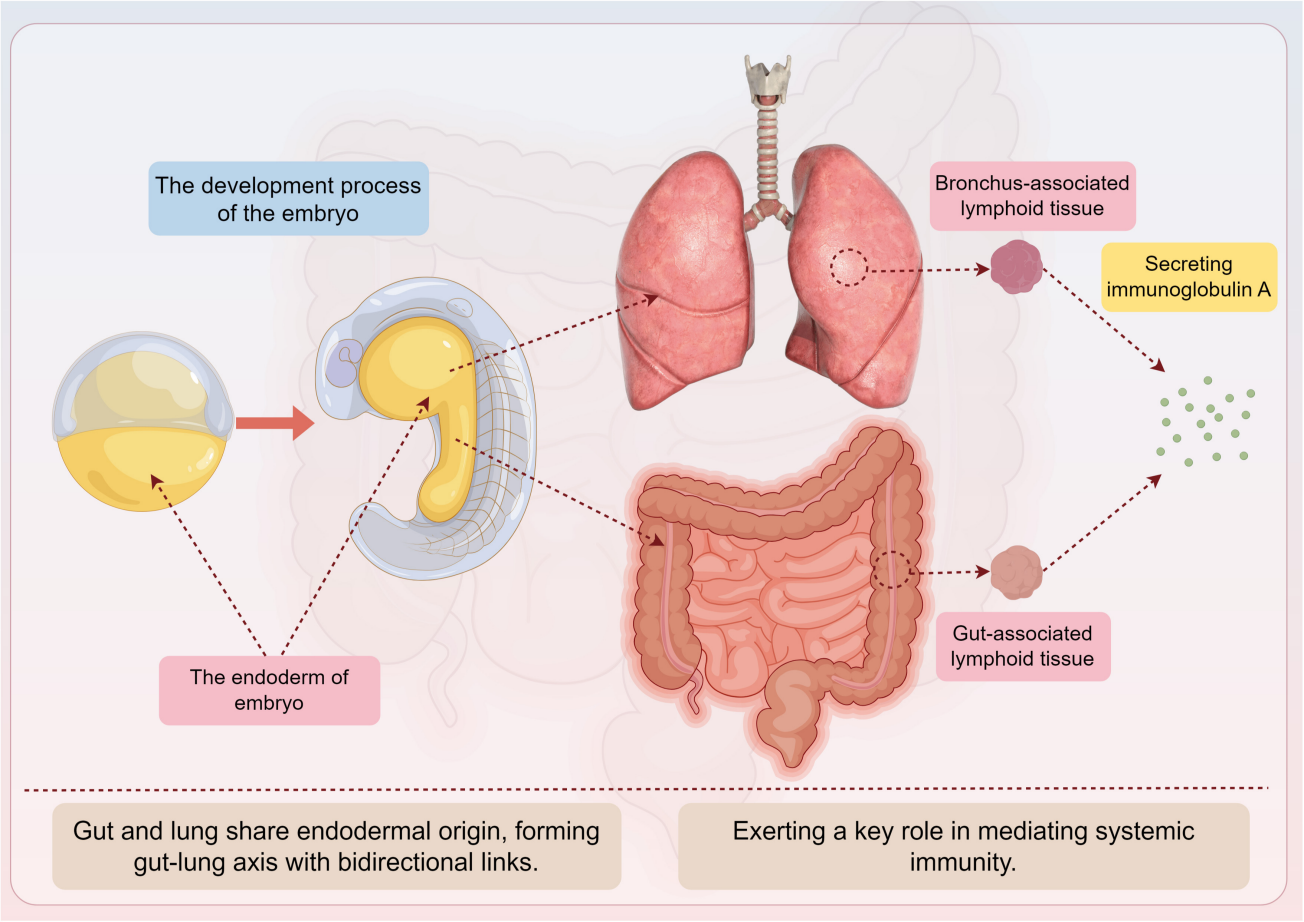
Anatomical and embryological links of the Gut-microbiota-lung Axis by Figdraw. This figure illustrates the common embryological origin of the gut and lung tissues and their anatomical features.

Understanding the physiological and immunological underpinnings of the Gut-microbiota-lung Axis is essential to grasp how these distant organs interact and maintain health (4). The gut and lungs share a common embryological origin, which forms the basis for their structural and functional similarities and the bidirectional communication between them (12) (Figure 1). By exploring these fundamental aspects, we can better comprehend the mechanisms through which gut microbiota affects lung cancer development and progression.

### 3.1 Anatomical and embryological links

The gut and lungs share a common endodermal origin during embryonic development, which lays the foundation for their structural and functional similarities and the bidirectional communication of the Gut-microbiota-lung Axis (4). Both the lung, trachea, respiratory epithelium, and gut originate from the endoderm (12). A study found that hyperactive Wnt signaling in lung progenitor cells expressing lung-specific genes can induce the differentiation of lung progenitor cells into gut cell types. The mucosal immune system, including gut-associated lymphoid tissue (GALT) and bronchus-associated lymphoid tissue (BALT), exerts a key role in mediating systemic immunity. Secreted immunoglobulin A (sIgA) produced by the mucosal immune system is a common molecular basis of mucosal immunity in different parts of the body and an important molecular mediator of the Gut-microbiota-lung Axis (4) (Figure 1). It is involved in the pathogenesis and progression of lung diseases such as Chronic obstructive pulmonary disease (COPD), asthma, and idiopathic pulmonary fibrosis, prevents the spread of pathogens in the body, and regulates the composition and function of gut microbiota. The poor outcome of germ-free mice exposed to acute infection and their susceptibility to allergic airway disease demonstrate the critical role of the gut microbiota in lung homeostasis and immunity (4). Researchers have also detected the expression of lung function protein pulmonary surfactant protein A in the gut tissue of patients with gut inflammation, further highlighting the similarity between the lung and gut (15).

### 3.2 Microbial and metabolic crosstalk

Gut microbiota-derived metabolites, such as SCFAs and bile acids, play a significant role in pulmonary inflammation. SCFAs, mainly propionate, acetate, and butyrate, are produced through the microbial fermentation of indigestible foods in the gastrointestinal tract (16). They maintain the proper functioning of the intestinal barrier, regulate glucose and lipid metabolism, alleviate oxidative stress and inflammation, and are considered main modulators of gut and lung immunity (17). The gut microbiota is the primary source of SCFAs influencing immune cells in the lamina propria and mesenteric lymph nodes (18). These cells then arrive in the respiratory system through circulation. For example, propionate produced in mice during a fiber-rich diet stimulates macrophages and dendritic cell progenitors, which can trigger phagocytosis without inducing Th2-mediated allergic airway inflammation (13, 19). SCFAs also affect hematopoietic precursor production in the bone marrow to maintain lung homeostasis and alleviate potential airway inflammation (20). In patients with emphysema, a positive correlation between higher fecal acetate levels and forced expiratory volume in the first second was observed (20). Exogenous acetate supplementation reduced alveolar destruction and pro-inflammatory cytokine production in mouse models of emphysema (21). In contrast, COPD patients showed a *Prevotella*-dominated gut type and lower SCFAs in feces, including acetic acid, isobutyric acid, and isovaleric acid (22). The severity of COPD patients was associated with reduced SCFAs concentrations in feces (23). Antibiotic-induced gut microbiota imbalance leading to SCFAs reduction aggravated the development of emphysema in mice (24). Gavage of acetate-producing *Bifidobacterium longum* subsp. *longum* was found to alleviate lung inflammation and butyrate depletion in the cecum of mice in a COPD model induced by 8 weeks of cigarette smoke exposure (23). Gut microbiota-derived SCFAs could directly or indirectly regulate the immune homeostasis of the lung, thereby alleviating the development of COPD.

Gut permeability and microbial translocation are drivers of systemic inflammation (25). Gut dysbiosis impairs epithelial barrier function and elicits a pro-inflammatory response (26). For instance, gut dysbiosis marked by a notable rise in Enterobacteriaceae activates TLR4 in the intestine, which elevates IL-1β levels in the peripheral circulation (25). This transmits inflammatory signals to the lungs and activates the NF-κB pathway, triggering oxidative stress and inflammation and contributing to lung pathology through the regulation of the intestinal barrier. ILC2s, ILC3s, and Th17 cells that migrate from the gut to the lungs have also been shown to impact respiratory immunity (25).

Gut-derived SCFAs shape pulmonary immunity, yet the lung microbiota itself is now recognized as an independent modulator of respiratory health. 16S rRNA profiling of bronchoalveolar-lavage fluid revealed that NSCLC tissue harbors a distinct luminal community enriched for *Streptococcus*, *Veillonella* and *Rothia*, with alpha-diversity inversely correlating with tumor stage (27, 28). Mechanistically, lung-colonizing *Streptococcus* spp. secrete peptidoglycan that activates NOD2 on alveolar macrophages, driving IL-1β-mediated MDSC recruitment and PD-L1 up-regulation within the tumor bed (29). Thus, local lung dysbiosis may synergize with gut-derived signals to amplify immunosuppression.

Tobacco smoke and COPD are major confounders that simultaneously remodel both gut and lung microbial compartments. In a COPD-NSCLC cohort, metagenomic sequencing showed smoke-related enrichment of *Prevotella* and *Porphyromonas* in sputum, while the same patients exhibited gut depletion of *Faecalibacterium* and reduced serum butyrate (30). Smoke-induced gut-barrier leakage elevated systemic LPS, which primed alveolar macrophages for enhanced IL-8 and MMP-12 release, thereby accelerating emphysema and creating a pro-metastatic niche (31). Conversely, 8-week smoking cessation partially restored gut-barrier integrity and re-balanced lung microbiota, supporting the reversibility of smoke-driven dysbiosis (23). Integrative analyses therefore suggest that COPD and smoking function as bidirectional amplifiers of gut–lung-axis perturbation, warranting stratification for microbiota-targeted trials in lung-cancer patients.

## 4 The mechanism of gut microbiota in the progression of lung cancer

**FIGURE 2:**
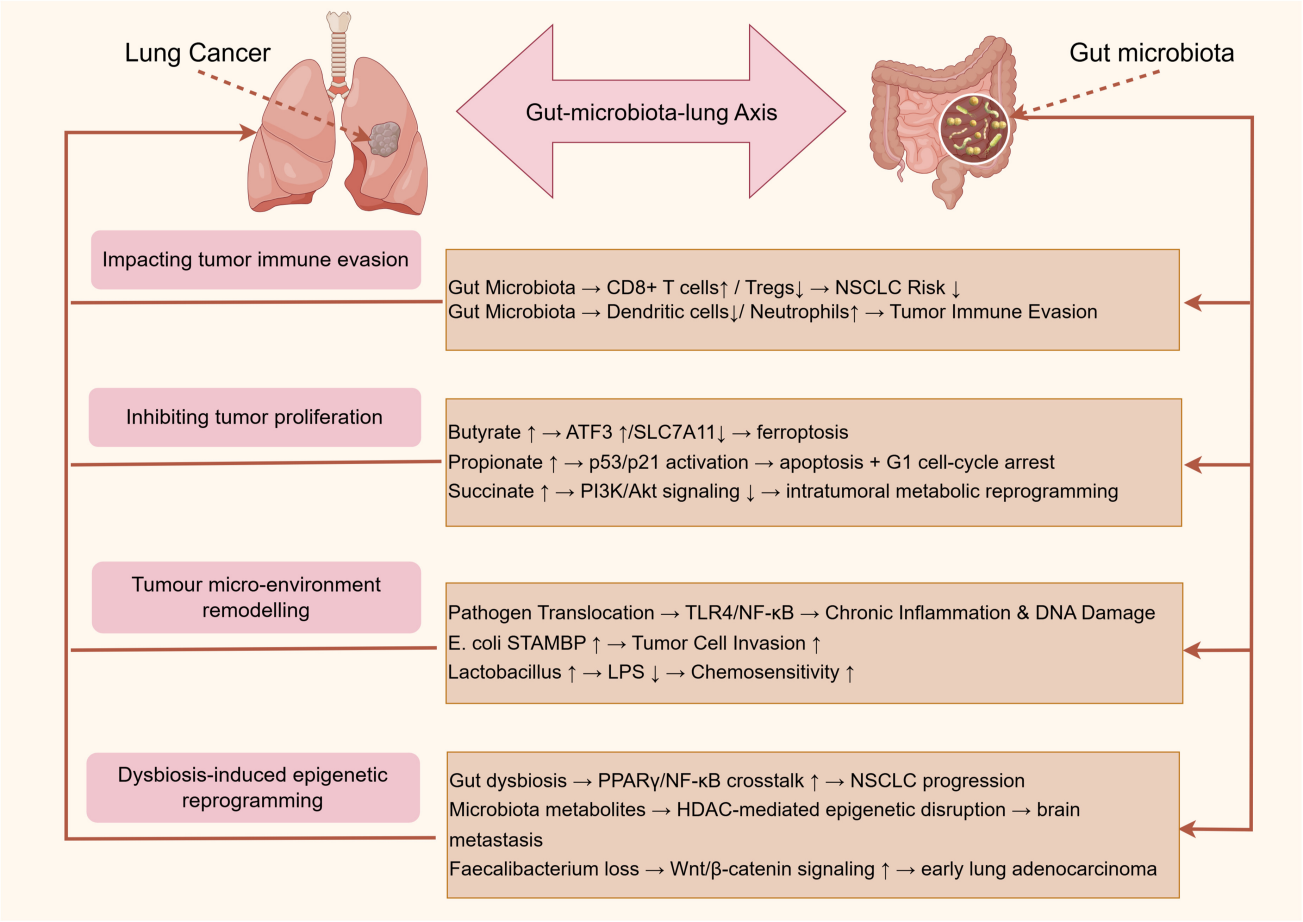
Mechanisms of gut microbiota in lung cancer progression by Figdraw.

Elucidating the complex interplay between gut microbiota and lung cancer progression reveals multiple mechanisms through which these microbial communities exert their influence (14). Emerging evidence highlights the role of gut microbiota in modulating systemic and local immune responses, producing metabolites with anticancer properties, and directly affecting the tumor microenvironment through microbial translocation (Table 1). Additionally, gut microbiota dysbiosis can lead to epigenetic modifications and the activation of oncogenic signaling pathways in lung cancer. Figure 2 proposes an integrated model that synthesizes current evidence into four, non-exclusive pathways: (i) systemic immunomodulation, (ii) microbial metabolite signaling, (iii) bacterial translocation and tumor micro-environment remodeling, and (iv) dysbiosis-induced epigenetic reprogramming.

**TABLE 1:** Studies on the mechanism of gut microbiota in lung cancer.

Flora/metabolites	Target (with sample size)	Mechanism of action	Role in lung cancer	References
*Bacteroides* spp.	CD8^+^ T cells, Tregs (*n* = 452 European GWAS)	Enhances CD8 + T-cell infiltration; suppresses Treg activity via immunomodulatory pathways	Reduces NSCLC risk by promoting antitumor immunity	Chen et al. (7)
*Faecalibacterium* spp.	Dendritic and neutrophil abundance (*n* = 452 GWAS)	Modulates dendritic cell and neutrophil abundance; reduces tumor immune evasion	Correlates inversely with NSCLC progression	Chen et al. (8)
Gut microbiota dysbiosis	SCLC risk (*n* = 2-sample MR, 24,000 Europeans)	No causal association observed in Mendelian randomization analysis	No significant impact on small cell lung cancer (SCLC) pathogenesis	Li et al. (32)
Butyrate	ATF3/SLC7A11 axis (*n* = 36 A/J male mice)	Synergizes with erastin to induce ferroptosis via ATF3 upregulation and SLC7A11 inhibition	Enhances NSCLC cell death; overcomes chemotherapy resistance	Bi et al. (35)
Propionate	p53/p21 pathway (*n* = 3 in-vit replicates; A549 and H1299)	Triggers apoptosis and cell cycle arrest via p53/p21 activation	Suppresses lung adenocarcinoma proliferation	Kim et al. (36)
*Akkermansia muciniphila*	PI3K/Akt signaling (*n* = 20 C57BL/6 mice)	Produces succinate to reprogram intratumoral metabolism; inhibits PI3K/Akt signaling	Suppresses NSCLC growth and metastasis	Zhu et al. (28)
Basil polysaccharide	Linoleic acid metabolism (*n* = 30 BALB/c nude mice)	Alters fecal metabolites (e.g., linoleic acid) to inhibit tumor proliferation	Synergizes with gefitinib to suppress NSCLC progression	Feng et al. (37)
SCFAs	Host metabolic status (*n* = 102 cachectic cancer patients)	Reduced levels in cachectic patients correlate with poor treatment response	Context-dependent efficacy; requires personalized approaches	Ubachs et al. (38)
*Klebsiella pneumoniae*	TLR4/NF-κB pathway (*n* = 32 human NSCLC tissues)	Promotes chronic inflammation and DNA damage via TLR4/NF-κB activation	Exacerbates NSCLC progression by inducing genomic instability	Dumont-Leblond et al. (39)
*Escherichia coli*	Circulating STAMBP (*n* = 45 tumor-bearing mice)	Elevates circulating STAMBP to enhance tumor cell invasion	Drives lung cancer metastasis through STAMBP-mediated signaling	Li et al. (40)
*Lactobacillus* spp.	Serum LPS (*n* = 77 Chinese NSCLC patients)	Reduces serum LPS levels; improves chemotherapy outcomes	Correlates with better prognosis in NSCLC patients	Zhao et al. (41)
*Streptococcus* spp.	Bronchoalveolar lavage microbiota (*n* = 56 NSCLC patients)	Bronchoalveolar lavage fluid microbiota linked to advanced NSCLC prognosis	Indicates bidirectional Gut-microbiota-lung Axis crosstalk in disease progression	Cheng et al. (27)
Diallyl trisulfide	PPARγ/NF-κB crosstalk (*n* = 30 A/J mice)	Restores gut microbial diversity; suppresses PPARγ/NF-κB crosstalk	Attenuates NSCLC by reducing inflammation and oxidative stress	Qu et al. (42)
Trimethylamine N-oxide (TMAO)	HDAC-mediated epigenetic axis (*n* = 68 patient metagenome)	Facilitates brain metastasis via HDAC-mediated epigenetic dysregulation	Promotes NSCLC metastasis to the brain	Liu et al. (43)
*Faecalibacterium* depletion	Wnt/β-catenin activation (*n* = 42 early-stage adenocarcinoma)	Correlates with aberrant Wnt/β-catenin activation in early-stage lung adenocarcinoma	Serves as a biomarker for early-stage NSCLC with oncogenic pathway dysregulation	Zeng et al. (44)
Gut microbiota dysbiosis	SCLC progression (*n* = 2-sample MR, 24 000 Europeans)	No significant association in Mendelian randomization analyses	Limited role in SCLC pathogenesis	Gong et al. (45)

ATF3, Activating Transcription Factor 3; HDAC, Histone Deacetylase; LPS, lipopolysaccharide; NSCLC, non-small cell lung cancer; PI3K/Akt, Phosphoinositide 3-Kinase/Protein Kinase B; PPARγ, Peroxisome Proliferator-Activated Receptor Gamma; SCFAs, short-chain fatty acids; SCLC, small cell lung cancer; STAMBP, signal transducing adaptor molecule-binding protein; Th17, T Helper 17 cells; TLR4, Toll-Like Receptor 4; TMAO, trimethylamine N-oxide; Tregs, regulatory T cells; Wnt/β-catenin, Wingless/Integrated-β-Catenin Signaling Pathway.

### 4.1 Immunomodulation and immune cell recruitment

Emerging evidence highlights the pivotal role of gut microbiota in modulating systemic and local immune responses, thereby influencing lung cancer progression. Mendelian randomization studies demonstrate causal links between gut microbiota composition and NSCLC risk, mediated by immune cell dynamics. For instance, Chen et al. (7) identified that *Bacteroides* and *Faecalibacterium* species inversely correlated with NSCLC risk, likely through enhancing CD8 + T cell infiltration and reducing regulatory T cell (Tregs) activity. Similarly, Chen et al. (8) revealed that gut microbiota dysbiosis altered the abundance of circulating dendritic cells and neutrophils, which directly impacted tumor immune evasion. However, Li et al. (32) found no causal association between gut microbiota and small cell lung cancer (SCLC) in Mendelian randomization study, suggesting histology-specific immunomodulatory mechanisms. Collectively, these studies underscore the gut microbiota’s capacity to shape antitumor immunity, though heterogeneity across lung cancer subtypes warrants further exploration.

While *Akkermansia muciniphila* enrichment is linked to enhanced CD8 + T-cell infiltration in European and North-American cohorts (33), the same taxon shows neutral or even negative associations in Asian populations receiving concurrent antibiotics (34). Geographic, dietary and concomitant medication factors therefore moderate the immunostimulatory potential of this species.

### 4.2 Metabolite-mediated anticancer effects

Short-chain fatty acids, particularly butyrate and propionate, derived from microbial fermentation of dietary fiber, exhibit direct anticancer effects. Bi et al. (35) demonstrated that butyrate synergized with erastin to induce ferroptosis in lung cancer cells by upregulating ATF3 and inhibiting SLC7A11, a glutathione synthesis regulator. Similarly, Kim et al. (36) showed propionate triggered apoptosis and cell cycle arrest in lung adenocarcinoma via p53/p21 activation. Conversely, Zhu et al. (28) revealed that *A. muciniphila*-produced metabolites, such as succinate, reprogrammed intratumoral metabolism to suppress NSCLC growth by downregulating PI3K/Akt signaling. These findings are corroborated by Feng et al. (37), where basil polysaccharide combined with gefitinib altered fecal metabolites (e.g., linoleic acid) to inhibit tumor proliferation. Nevertheless, Ubachs et al. (38) reported reduced SCFA levels in cachectic lung cancer patients, implying that metabolite efficacy may depend on host metabolic status.

Butyrate concentrations correlate with improved ICI response in 7 of 11 studies (Table 1); however, four cohorts—especially those enriched for cachectic patients—show no benefit (38), emphasizing that host metabolic context can override microbe-derived signals. Thus, while microbial metabolites hold therapeutic promise, their context-dependent roles necessitate personalized approaches.

### 4.3 Microbial translocation and tumor microenvironment remodeling

Gut microbiota-derived components, including lipopolysaccharides (LPS) and live bacteria, may translocate to the lung, directly influencing carcinogenesis. Dumont-Leblond et al. (39) detected enteric pathogens like *Klebsiella pneumoniae* in NSCLC tissues, which promoted chronic inflammation and DNA damage via TLR4/NF-κB activation. Li et al. (40) further identified gut Escherichia coli as a key mediator of lung cancer progression, elevating circulating signal transducing adaptor molecule-binding protein (STAMBP) levels to enhance tumor cell invasion. Conversely, Zhao et al. (41) observed that Lactobacillus enrichment in the gut correlated with reduced serum LPS and improved chemotherapy outcomes. Notably, Cheng et al. (27) linked bronchoalveolar lavage fluid microbiota (e.g., Streptococcus) to advanced NSCLC prognosis, suggesting bidirectional Gut-microbiota-lung Axis crosstalk. These studies highlight the dual role of microbial translocation—pathogenic taxa exacerbate malignancy, while commensals may confer protection. Detection of live gut-derived bacteria in lung tumors is reported in fewer than 15% of resected NSCLC specimens; thus, direct bacterial colonization is likely relevant to a molecular subtype rather than to lung cancer universally (39).

### 4.4 Dysbiosis-driven epigenetic and signaling pathway alterations

Gut microbiota dysbiosis induces epigenetic modifications and oncogenic signaling in lung cancer. Qu et al. (42) found that diallyl trisulfide attenuated NSCLC by restoring gut microbial diversity and suppressing PPARγ/NF-κB crosstalk. Liu et al. (43) demonstrated that gut microbiota metabolites (e.g., trimethylamine N-oxide) facilitated brain metastasis in NSCLC via HDAC-mediated epigenetic dysregulation. Additionally, Zeng et al. (44) identified *Faecalibacterium* depletion as a marker of aberrant Wnt/β-catenin activation in early-stage lung adenocarcinoma. However, Gong et al. (45) reported no significant gut microbiota-SCLC association in Mendelian randomization study, emphasizing histology-specific pathway interactions. Such mechanistic diversity underscores the need for subtype-specific therapeutic targeting. *Faecalibacterium prausnitzii* depletion consistently associates with Wnt/β-catenin activation in early-stage adenocarcinoma (44), yet Mendelian randomization studies fail to support a causal role for this taxon in SCLC, underlining histology-specific pathways (32).

## 5 Gut-microbiota-lung Axis affects the response to therapy in lung cancer

Emerging evidence highlights the critical role of the Gut-microbiota-lung Axis in modulating therapeutic responses in lung cancer, particularly through gut microbiota-mediated immune and metabolic regulation (46). This section evaluates the impact of gut microbiota on treatment efficacy and toxicity across different therapeutic modalities, with a focus on ICIs, chemotherapy, and combination therapies (Table 2).

**TABLE 2:** Research on the influence of the Gut-microbiota-lung Axis on the treatment response of lung cancer.

Flora/metabolites	Study types (with n)	Treatment measures	Mechanism of action	References
Antibiotics-induced dysbiosis	Retrospective cohort (*n* = 60 NSCLC)	Immune checkpoint inhibitors	Reduced systemic immunity via depletion of immunostimulatory taxa (e.g., *Akkermansia muciniphila*)	Derosa et al. (47)
Antibiotics	Observational study (*n* = 74 NSCLC)	Anti-PD-1 therapy	Over 70% reduction in OS; impaired CD8 + T cell activation	Hamada et al. (48)
*Faecalibacterium prausnitzii*	Phase-I trial (*n* = 38 enrolled)	ICIs (anti-PD-1/PD-L1)	Enhanced dendritic cell activation and CD8 + T cell infiltration; increased ORR (52% vs. 28%)	Bredon et al. (33)
Butyrate (SCFAs)	Metabolomic analysis (*n* = 49 Italian patients)	Anti-PD-1 therapy	Higher fecal butyrate levels correlated with T cell activation in responders	Botticelli et al. (49)
*Clostridium butyricum*	Randomized trial (*n* = 42 Japanese)	ICIs + PPIs	Restored ICI efficacy by compensating for butyrate deficiency; improved median PFS (6.1 vs. 3.4 months)	Tomita et al. (50)
*Bifidobacterium*	Animal model (*n* = 18 C57BL/6)	Anti-PD-1 therapy	Extracellular vesicles synergized with ICIs to suppress tumor growth via immune modulation	Preet et al. (63)
Gut microbiota diversity	Prospective cohort (*n* = 74 European)	Nivolumab (anti-PD-1)	No significant association between baseline microbiota and survival outcomes	Ouaknine Krief et al. (34)
Serum butyrate	Prospective cohort (*n* = 94 Chinese)	Platinum-based chemotherapy	Higher serum butyrate levels linked to improved ORR (68% vs. 42%) via apoptosis induction	Chen et al. (55)
Antibiotics	Retrospective cohort (*n* = 153 Chinese)	Chemoimmunotherapy	Lower ORR (32% vs. 51%) and higher grade ≥3 AEs (45% vs. 28%)	Deng et al. (56)
Pemetrexed	Pre-clinical PDX model (*n* = 12 mice)	Chemotherapy	Disrupted gut microbiota diversity; exacerbated intestinal inflammation	Pensec et al. (57)
BFHY herbal formula	Animal model (*n* = 24 BALB/c)	Cisplatin chemotherapy	Attenuated intestinal toxicity via *Lactobacillus* enrichment and anti-inflammatory effects	Feng et al. (58)
*Bacteroides vulgatus*	Prospective cohort (*n* = 112 NSCLC)	Chemoradiotherapy	Reduced radiation-induced pneumonitis risk (HR = 0.47)	Qiu et al. (59)
Antibiotics-induced dysbiosis	Real-world analysis (*n* = 174 Japanese)	Platinum-pembrolizumab	Lower ORR (29% vs. 44%) and shorter median OS (12.1 vs. 18.9 months)	Tamura et al. (60)
Fecal microbiota transplantation (FMT)	Pre-clinical murine model (*n* = 24 LLC-bearing mice)	Chemoimmunotherapy	Enriched *Bifidobacterium* and *Akkermansia*; enhanced tumor control	Wang et al. (61)
Probiotics	Phase-II trial (*n* = 96 Chinese)	Chemoimmunotherapy	Improved ORR (58% vs. 41%) and reduced gastrointestinal AEs (22% vs. 45%)	Xia et al. (62)
Probiotics	Randomized trial (*n* = 200 Japanese)	ICIs ± chemotherapy	No significant survival benefit observed; strain-dependent variability	Morita et al. (64)

ORR, objective response rate; OS, overall survival; PFS, progression-free survival; PPIs, proton pump inhibitors; SCFAs, short-chain fatty acids; AEs, adverse events; PDX, Patient-Derived Xenograft.

### 5.1 ICIs

The gut microbiota significantly influences ICIs efficacy by shaping systemic and tumor microenvironment immunity. Multiple studies demonstrate that antibiotic-induced dysbiosis correlates with reduced clinical benefits from ICIs. For instance, Derosa et al. (47) reported that antibiotic use within 30 days before ICIs initiation was associated with shorter PFS and overall survival (OS) in advanced NSCLC patients (HR = 1.5, *p* = 0.001). Similarly, Hamada et al. (48) found that antibiotic exposure reduced OS by over 70% in NSCLC patients receiving anti-PD-1 therapy, likely due to depletion of immunostimulatory taxa like *Akkermansia muciniphila*. Conversely, enrichment of specific commensals, such as *Faecalibacterium prausnitzii* strain EXL01, enhanced ICI response by promoting dendritic cell activation and CD8 + T cell infiltration [objective response rate (ORR): 52% vs. 28% in controls, *p* = 0.02] (33).

Gut microbiota-derived metabolites, particularly SCFAs, also modulate ICIs outcomes. Botticelli et al. (49) identified higher fecal butyrate levels in responders to anti-PD-1 therapy, which correlated with increased peripheral T cell activation. A randomized trial by Tomita et al. (50) further showed that *Clostridium butyricum* supplementation restored ICIs efficacy in patients receiving proton pump inhibitors (PPIs), likely by compensating for butyrate deficiency (median PFS: 6.1 vs. 3.4 months, *p* = 0.03). Conflicting evidence surrounds *Bifidobacterium*’s clinical relevance, as high baseline *B. breve* abundance predicted longer PFS in Asian NSCLC patients receiving anti-PD-1 plus chemotherapy (51), yet a European cohort found no genus-level survival benefit after adjustment for antibiotics, PPIs and tumor mutational burden (34). These discordant outcomes likely reflect strain-specific effects, since only *B. breve* was protective, together with higher fiber intake and fecal butyrate in the Asian population that supports *Bifidobacterium* colonization (52), frequent PPI use in Europe that lowers gastric pH and impairs engraftment (53), and host genetic factors such as the East-Asian-enriched HLA-B allele that enhances mucosal IgA targeting of *Bifidobacterium* antigens (54). Such context emphasizes the need for strain-resolved, diet-adjusted and medication-controlled analyses before *Bifidobacterium* biomarker implementation.

### 5.2 Chemotherapy

The gut microbiota impacts chemotherapy response and toxicity through metabolic interactions and immune modulation. Chen et al. (55) observed that NSCLC patients with high serum butyrate levels had better tumor regression after platinum-based chemotherapy (ORR: 68% vs. 42%, *p* = 0.01), likely via SCFA-induced apoptosis of cancer cells. Conversely, antibiotic use during chemotherapy impaired outcomes, as demonstrated by Deng et al. (56), where NSCLC patients receiving antibiotics had lower ORR (32% vs. 51%, *p* = 0.02) and higher rates of grade ≥3 adverse events (AEs) (45% vs. 28%, *p* = 0.03). Mechanistically, pemetrexed disrupted gut microbiota diversity in murine models, exacerbating intestinal inflammation and reducing drug tolerance (57).

Notably, gut microbiota modulation may ameliorate chemotherapy toxicity. Feng et al. (58) reported that a herbal formula (BFHY) attenuated cisplatin-induced intestinal damage in mice by restoring Lactobacillus abundance and suppressing pro-inflammatory cytokines (e.g., IL-6, TNF-α). Similarly, Qiu et al. (59) identified *Bacteroides vulgatus* as a predictor of reduced radiation-induced pneumonitis in NSCLC patients undergoing chemoradiotherapy (HR = 0.47, *p* = 0.01). These findings suggest microbiota-targeted interventions could optimize chemotherapy safety.

### 5.3 Combination therapies

The gut microbiota’s role in chemoimmunotherapy (e.g., platinum-pemetrexed plus ICIs) is increasingly recognized. Tamura et al. (60) found that antibiotic-induced dysbiosis diminished the efficacy of platinum-pembrolizumab in NSCLC, with lower ORR (29% vs. 44%, *p* = 0.04) and shorter median OS (12.1 vs. 18.9 months, *p* = 0.01). Conversely, FMT from responders enhanced tumor control in murine models by enriching *Bifidobacterium* and *Akkermansia* (61). A phase II trial by Xia et al. (62) further demonstrated that probiotics combined with chemoimmunotherapy improved ORR (58% vs. 41%, *p* = 0.04) and reduced gastrointestinal AEs (22% vs. 45%, *p* = 0.02) in advanced NSCLC patients.

Despite these advances, conflicting data exist. For example, while Preet et al. (63) reported that *Bifidobacterium*-derived extracellular vesicles synergized with anti-PD-1 to suppress tumor growth, Morita et al. (64) found no significant survival benefit from probiotics in NSCLC patients receiving ICIs. These discrepancies may stem from differences in probiotic strains, dosing regimens, or host genetic factors.

## 6 Therapeutic interventions targeting the Gut-microbiota-lung Axis

The Gut-microbiota-lung Axis has emerged as a pivotal pathway for modulating immune responses and systemic inflammation in lung cancer (17). Emerging therapeutic strategies targeting this axis focus on reshaping gut microbiota composition (Table 3), regulating microbial metabolites, and enhancing ICIs efficacy (65).

**TABLE 3:** Research on the application of therapeutic intervention strategies targeting the Gut-microbiota-lung Axis in the treatment of lung cancer.

Intervention strategies	Targets	Study types (with sample size)	Therapeutic effect	References
Probiotics (CBM588)	Gut microbiota, T-cell infiltration	Prospective clinical trial (*n* = 40 Japanese patients)	Improved OS and ORR in lung cancer patients receiving chemoimmunotherapy	Tomita et al., (66);
Probiotics (CBM588)	Gut microbiota, T-cell infiltration	Prospective clinical trial (*n* = 42 Japanese patients)	Confirmed survival benefit with CBM588 plus chemo-immunotherapy	Tomita et al., (67)
Generic probiotics	Gut microbiota diversity	Retrospective cohort (*n* = 1 841 multi-cancer patients, 229 NSCLC)	No significant survival benefit in ICI-treated patients; strain-dependent variability	Wan et al. (68)
*Bifidobacterium breve*	Anti-PD-1 efficacy	Biomarker analysis (*n* = 126 Chinese NSCLC patients)	Predicted improved outcomes in NSCLC patients on anti-PD-1 + chemotherapy	Zhao et al., (51)
Dietary interventions	NF-κB-driven inflammation	Pre-clinical murine model (*n* = 30 A/J mice)	Cigarette smoke-induced dysbiosis exacerbated lung cancer progression	Qu et al., (69)
Ginseng polysaccharides	Kynurenine/tryptophan ratio, CD8 + T cells	Randomized controlled trial (*n* = 68 Chinese patients)	Enhanced anti-PD-1 efficacy via immune modulation	Huang et al., (52)
Theabrownin	PI3K/Akt/mTOR pathway	Murine colorectal model (*n* = 20 C57BL/6 mice)	Suppressed tumorigenesis via pathway inhibition and microbiota modulation	Leung et al., (70)
Xihuang Pill	VEGF, HIF-1α, gut microbiota	Pre-clinical + clinical (*n* = 60 mice; *n* = 28 patient metagenome)	Synergized with anlotinib to suppress angiogenesis and tumor growth	Cao et al.,(71)
BuFeiXiaoJiYin	NLRP3 inflammasome, Treg/Th17 balance	Murine lung cancer model (*n* = 24 BALB/c mice)	Ameliorated inflammation and restored gut microbiota equilibrium	Jiang et al., (72)
EGCG	STAT1/SLC7A11 pathway	Obesity-driven murine model (*n* = 30 C57BL/6 mice)	Alleviated lung cancer progression via metabolic and microbiota regulation	Li et al., (73)
FMT (Alzheimer’s model feces)	*Akkermansia*, Enterobacteriaceae	Pre-clinical murine model (*n* = 20 C57BL/6 mice)	Accelerated lung tumor growth via pro-inflammatory microbiota shift	Bi et al., (74)
Postbiotics (JK5G)	Immune-related adverse events (irAEs)	Randomized controlled trial (*n* = 60 Chinese NSCLC patients)	Reduced irAEs in NSCLC patients via microbiota modulation	Chen et al., (75)
*Helicobacter pylori* screening	ICI efficacy	Retrospective cohort (*n* = 404 melanoma patients, validation lung subset *n* = 97)	Seropositivity correlated with reduced OS in melanoma patients on ICIs	Tonneau et al., (77)
Proton pump inhibitors (PPIs)	Gastric pH, microbiota composition	*Post hoc* clinical analysis (*n* = 692 IMpower150 NSCLC patients)	Attenuated atezolizumab efficacy in NSCLC patients	Hopkins et al., (53)
Metformin	*Akkermansia muciniphila*, butyrate	Pre-clinical murine model (*n* = 18 C57BL/6 mice)	Enhanced anti-PD-L1 activity via microbiota regulation	Zhao et al., (78)
Synbiotics (Inulin + Sintilimab)	Gut microbiota-derived T-cell immunity	Murine lung adenocarcinoma model (*n* = 18 LL/2 mice)	Suppressed tumor growth by enhancing T-cell activity	Yan et al., (79)
Engineered *Diaphorobacter nitroreducens*	ROS-mediated apoptosis	Pre-clinical murine model (*n* = 15 LLC mice)	Synergized with oxaliplatin to reduce lung adenocarcinoma burden	Ni et al., (80)

ORR, objective response rate; OS, overall survival; PFS, progression-free survival; PPIs, proton pump inhibitors; SCFAs, short-chain fatty acids; AEs, adverse events; PDX, Patient-Derived Xenograft; BFHY, BFHY herbal formula; HR, Hazard Ratio; NLRP3, NLR Family Pyrin Domain Containing 3; Treg/Th17, regulatory T cells/T Helper 17 cells; HIF-1α, Hypoxia-Inducible Factor 1-Alpha; VEGF, Vascular Endothelial Growth Factor; STAT1, Signal Transducer and Activator of Transcription 1; SLC7A11, Solute Carrier Family 7 Member 11; PI3K/Akt, Phosphoinositide 3-Kinase/Protein Kinase B; TMAO, trimethylamine N-oxide; Tregs, regulatory T cells; TLR4, Toll-Like Receptor 4; FMT, fecal microbiota transplantation; ICI, immune checkpoint inhibitor.

### 6.1 Probiotics and microbial modulation

Probiotics, particularly *Clostridium butyricum* (CBM588), have demonstrated promising immunomodulatory effects. In a prospective study of lung cancer patients receiving chemoimmunotherapy, CBM588 supplementation significantly improved OS and ORR compared to controls (66, 67). Whether these effects reflect prognostic enrichment or true predictive utility remains unresolved. Mechanistically, CBM588 enhances butyrate production, which promotes T-cell infiltration and reduces immunosuppressive cytokines like IL-10 and TGF-β (66). However, inconsistencies exist: while Tomita et al. (66) reported prolonged survival in patients receiving CBM588, Wan et al. (68) found no significant survival benefit with generic probiotics in ICIs-treated cohorts, suggesting strain-specific effects and the importance of butyrogenic species. Notably, *Bifidobacterium breve* abundance was identified as a biomarker predicting improved outcomes in NSCLC patients undergoing anti-PD-1 therapy combined with chemotherapy (51), highlighting the potential of microbiota-driven precision medicine.

*Post hoc* analyses of two prospective Japanese cohorts (*n* = 40 and *n* = 42) showed that baseline abundance of *Faecalibacterium prausnitzii* ≥1.2% was an independent prognostic factor for longer OS (HR 0.48, 95% CI 0.26–0.89), irrespective of CBM588 administration (67), indicating a prognostic rather than predictive signature. Conversely, in the phase-I study of *F. prausnitzii* strain EXL01, only recipients who achieved ≥2-fold post-supplementation expansion of the strain derived significant ORR benefit (52% vs. 28% in non-expanders, *p* = 0.02), supporting a predictive biomarker role (33). Distinguishing prognostic from predictive value therefore requires longitudinal sampling during intervention; static baseline taxon abundance alone is insufficient to claim predictive utility.

### 6.2 Dietary interventions and microbial metabolites

Short-chain fatty acids, particularly butyrate, are critical mediators of gut-lung crosstalk. Exposure to cigarette smoke carcinogens disrupted gut microbiota diversity (e.g., increased Firmicutes/*Bacteroidetes* ratio) and exacerbated lung cancer progression via NF-κB-driven inflammation (69). Conversely, dietary interventions such as ginseng polysaccharides altered the gut microbiota and kynurenine/tryptophan ratio, enhancing anti-PD-1 efficacy by increasing CD8 + T-cell activity (52). Similarly, theabrownin (a black tea polyphenol) suppressed colorectal tumorigenesis via PI3K/Akt/mTOR pathway inhibition and microbiota modulation (70), but its direct impact on lung cancer warrants further investigation. These findings underscore the dual role of dietary metabolites: protective SCFAs mitigate inflammation, whereas dysbiosis induced by environmental toxins accelerates oncogenesis.

### 6.3 Herbal medicine and natural compounds

Traditional Chinese medicine (TCM) formulations, such as Xihuang Pill and Qingfei Mixture, synergize with chemotherapy by modulating gut microbiota and angiogenesis pathways. Xihuang Pill increased *Lactobacillus* and *Bifidobacterium* abundance, downregulating VEGF and HIF-1α expression in tumor microenvironments (71). Similarly, Bu Fei Xiao Ji Yin ameliorated NLRP3-mediated inflammation in lung cancer mice by restoring gut microbiota balance and enhancing Treg/Th17 equilibrium (72). However, variability in TCM composition and bioavailability poses challenges in standardizing clinical outcomes. For instance, while EGCG (epigallocatechin gallate) alleviated obesity-driven lung cancer via STAT1/SLC7A11 signaling (73), its low bioavailability necessitates further optimization for therapeutic use.

### 6.4 FMT and microbial reprogramming

Fecal microbiota transplantation is the most direct strategy to re-engineer the entire gut ecosystem and has moved from *Clostridioides difficile* therapy to oncology trials. In two independent pre-clinical lung-cancer models, FMT from ICI-responding donors restored anti-PD-1 efficacy and tripled median survival after antibiotic-induced dysbiosis (61). Metagenomic tracking showed engraftment of *Bifidobacterium longum* and *Akkermansia muciniphila* and a parallel expansion of tumor-infiltrating CD8 + T cells, indicating that FMT can reconstitute both immunostimulatory taxa and systemic anti-tumor immunity. Conversely, FMT from Alzheimer’s disease mice accelerated urethane-driven lung tumors through selective loss of *Akkermansia* and overgrowth of LPS-high Enterobacteriaceae (74), underscoring the importance of donor screening.

A first-in-human phase I study (NCT05122546) enrolled 12 refractory NSCLC patients who received a single naso-jejunal FMT from a verified ICI-responder; 3 patients achieved stable disease and one partial response, with no ≥grade-2 adverse events (75). Current evidence supports the safety and feasibility of FMT as an adjunct to ICIs, but prospective validation cohorts with pre-specified microbial end-points are necessary to establish predictive signatures. Although objective response rates remain modest, FMT was safe and led to durable engraftment of butyrate producers for ≥12 weeks. Ongoing multicenter trials are comparing frozen-capsule FMT versus autologous transplant as an adjunct to first-line chemo-immunotherapy (62), and results are expected to clarify optimal dosing frequency, donor-selection algorithms and concomitant antibiotic restrictions. Compared with single-strain probiotics, FMT offers the theoretical advantage of transferring a complete, self-sustaining microbial network; however, standardization of donor material, preparation protocols and long-term safety surveillance remain unresolved (76). Until phase-II efficacy data are available, FMT should be restricted to clinical trial settings with rigorous microbiological and immunological monitoring.

### 6.5 ICIs and microbiota interactions

The gut microbiota profoundly influences ICIs efficacy. *Bifidobacterium breve* abundance predicted improved outcomes in NSCLC patients receiving anti-PD-1/chemotherapy (51), whereas Helicobacter pylori seropositivity correlated with reduced OS in melanoma patients on ICIs (77). Pharmacomicrobiomics studies revealed that proton pump inhibitors (PPIs) attenuated atezolizumab efficacy by altering gastric pH and microbiota composition (53). Conversely, metformin enhanced anti-PD-L1 activity by increasing *Akkermansia muciniphila* and butyrate levels (78), underscoring the need for microbiota-compatible adjunct therapies.

Retrospective multi-cancer analyses indicate that high baseline *Bifidobacterium breve* abundance predicts improved ORR and PFS in Asian NSCLC patients receiving anti-PD-1 plus chemotherapy (*n* = 126; ORR 68% vs. 41%, *p* < 0.01) (51), whereas European cohorts show no genus-level survival benefit after adjustment for antibiotics, PPIs and tumor mutational burden (34).

These geographically divergent results underscore that microbial biomarkers may exhibit population-specific predictive performance, necessitating external validation before clinical implementation.

### 6.6 Emerging strategies: synbiotics and engineered microbes

Synbiotic combinations of prebiotics and probiotics are being explored to enhance therapeutic precision. For example, prebiotics (e.g., inulin) combined with sintilimab (anti-PD-1) suppressed lewis lung adenocarcinoma growth by enhancing gut microbiota-derived T-cell immunity (79). Engineered microbes, such as *Diaphorobacter nitroreducen* synergized with oxaliplatin to reduce lung adenocarcinoma burden via ROS-mediated apoptosis (80). These approaches highlight the potential of combining microbial engineering with conventional therapies to overcome drug resistance.

## 7 Technological advances in Gut-microbiota-lung Axis research

Advancements in scientific technology have revolutionized the study of the Gut-microbiota-lung Axis, offering innovative tools to investigate its complex mechanisms (20). Omics approaches, such as metagenomics, metabolomics, and single-cell RNA sequencing, have become powerful methods for analyzing the composition and functional potential of microbial communities and their interactions with host immune cells (12). Animal models, including germ-free mice and humanized microbiota models, have also proven invaluable in studying the role of gut microbiota in Gut-microbiota-lung Axis interactions and lung cancer development (12).

### 7.1 Omics approaches

Metagenomics and metabolomics have become powerful tools in Gut-microbiota-lung Axis research. Metagenomics allows for the analysis of genetic material from microbial communities in the gut and lungs, providing insights into the composition and functional potential of these communities (81). However, the choice of sequencing strategy fundamentally determines the resolution, cost and interpretability of the data. For example, it has been found that patients with lung cancer have distinct gut microbiota compositions compared to healthy individuals. Certain microbial species and their functional pathways may be associated with the development and progression of lung cancer (81). Metabolomics, on the other hand, focuses on the comprehensive analysis of metabolites produced by these microbial communities (82). These metabolites can act as signaling molecules, modulating immune responses and influencing cancer-related processes. For instance, SCFAs, produced by gut microbiota through the fermentation of dietary fiber, have been shown to have immunomodulatory effects and may play a role in regulating lung immunity and inflammation (82). Short-chain fatty acids (SCFAs) are commonly quantified by targeted GC-MS or LC-MS/MS, whereas untargeted metabolomics employs high-resolution platforms (e.g., UHPLC-QTOF-MS) to discover novel microbial metabolites. Studies have found that SCFAs can affect the function of immune cells in the lungs, such as macrophages and T cells, thereby potentially influencing the tumor microenvironment in lung cancer (83, 84).

However, there are some differences in the findings of different studies. Some research suggests that specific bacterial species or metabolites are associated with an increased risk of lung cancer, while others indicate that they may have protective effects (12, 17). For example, certain studies have reported that the abundance of specific bacteria in the gut, such as *Firmicutes* and Bacteroidetes, is altered in lung cancer patients, but the exact relationship and underlying mechanisms remain to be fully elucidated (85–87). This inconsistency may be due to differences in study populations, methodologies, and other factors. Therefore, further large-scale, well-designed studies are needed to clarify the specific roles of these microbial components and their metabolites in lung cancer development.

Single-cell RNA sequencing (scRNA-seq) has revolutionized our understanding of immune-microbial interactions in the Gut-microbiota-lung Axis (65). This technology enables the analysis of gene expression at the single-cell level, providing a highly detailed view of the heterogeneity and functional states of immune cells in the gut and lungs (88). For example, scRNA-seq has revealed diverse subsets of immune cells, such as T cells, B cells, and macrophages, and their unique transcriptional profiles in response to microbial stimuli (89). By analyzing these transcriptional changes, researchers can gain insights into how gut microbiota influences the differentiation, activation, and function of immune cells, and how these immune cells, in turn, affect lung cancer development and immune responses (90). Some studies have shown that specific gut microbiota compositions can modulate the tumor-infiltrating immune cell landscape in the lungs, thereby influencing the efficacy of immunotherapy for lung cancer (29, 54). For instance, the presence of certain bacteria in the gut has been associated with increased numbers of cytotoxic T cells and natural killer cells in the lung tumor microenvironment, which may enhance the response to immune checkpoint inhibitors (8, 91).

Nevertheless, there are also discrepancies in the results of different studies. The specific types of immune cells and their functional states influenced by gut microbiota may vary depending on factors such as the composition and function of the microbiota, the genetic background of the host, and the stage of lung cancer (12, 92). Therefore, it is necessary to conduct more in-depth and comprehensive studies to fully understand the complex interactions between gut microbiota and immune cells in the context of lung cancer.

### 7.2 Animal models

Germ-free (GF) mice, which are raised in a sterile environment and lack exposure to microbiota, have been invaluable in studying the role of gut microbiota in Gut-microbiota-lung Axis interactions (93, 94). By colonizing GF mice with specific microbial communities, researchers can investigate the effects of these microbes on immune system development, lung function, and cancer-related processes (95). For example, studies have shown that the absence of gut microbiota in GF mice leads to impaired immune system development and function, and increased susceptibility to respiratory infections and lung cancer. When these mice are colonized with a normal gut microbiota, their immune systems and lung health are partially restored (96). This suggests that gut microbiota plays a crucial role in maintaining immune homeostasis and protecting against lung diseases.

Humanized microbiota models, which involve transferring human gut microbiota into GF mice or other animal models, further enable the study of the specific effects of human microbiota on Gut-microbiota-lung Axis interactions and lung cancer development (92). These models provide a more clinically relevant system for investigating the mechanistic links between gut microbiota and lung cancer, and for testing potential therapeutic interventions targeting the Gut-microbiota-lung Axis (97). For instance, researchers can use humanized microbiota models to evaluate the impact of specific probiotics or prebiotics on the composition and function of gut microbiota, and subsequently assess their effects on immune responses and tumor growth in the lungs (98).

However, there are also some limitations and differences in the results obtained from different animal models. The gut microbiota of mice differs from that of humans in terms of composition and function, which may affect the translatability of findings to human clinical settings (99). Additionally, the complexity of the Gut-microbiota-lung Axis and the multiple factors involved in its regulation make it challenging to fully recapitulate the human disease conditions in animal models (100). Therefore, it is important to carefully interpret the results from animal studies and to validate them in human clinical studies whenever possible.

## 8 Challenges and future directions

The manipulation of the gut microbiota holds promise for the treatment of lung cancer, however, the lack of standardized protocols poses a significant challenge (14). Currently, interventions such as FMT, probiotics, and prebiotics are being explored. But the preparation, administration, and quality control of these interventions vary across studies (14). For example, FMT can be administered via different routes, such as nasogastric tubes or capsules, and the donor selection criteria and fecal processing methods also differ. These variations make it difficult to compare results across studies and to translate findings into clinical practice (101). Li et al. (76) demonstrated that FMT could improve the efficacy of immunotherapy in lung cancer patients, but the long-term safety and optimal dosing regimens remain unclear. Similarly, probiotic and prebiotic interventions also lack standardized protocols. Different strains and doses of probiotics may have varying effects on the gut microbiota and immune system (102). Therefore, establishing standardized protocols for microbiota manipulation is crucial for advancing clinical applications.

The gut microbiome varies significantly among individuals due to factors such as genetics, diet, and lifestyle (103). This heterogeneity necessitates the development of personalized microbiome-based therapies for lung cancer patients (20). However, achieving personalization is challenging. First, a comprehensive understanding of the relationship between the gut microbiome and individual clinical outcomes is required (20). Studies have shown that certain microbial signatures are associated with better responses to immunotherapy, but these signatures may not be universal. For instance, some research indicates that a higher abundance of specific bacteria, such as *Akkermansia muciniphila*, is linked to improved immunotherapy responses, while other studies report different associations (104, 105). Second, the dynamic nature of the gut microbiome further complicates personalization. The microbiome can change over time due to factors like diet and medication use. Therefore, developing personalized therapies requires continuous monitoring and adjustment of the microbiome (90). Additionally, integrating microbiome data with other clinical and molecular data is necessary to create more precise treatment plans (106). Despite these challenges, personalized microbiome-based therapies offer a potential avenue for improving lung cancer treatment outcomes.

The Gut-microbiota-lung Axis involves two-way communication between the gut and lungs, and the lung microbiota plays a crucial role in this process (107). However, the exact role of the lung microbiota in Gut-microbiota-lung Axis dynamics remains poorly understood. Some studies suggest that the lung microbiota influences systemic immunity and inflammation, which in turn affect gut microbiota composition and function (108). For example, Dora et al. (105) found that alterations in the lung microbiota could impact the gut immune system through immune cell trafficking and cytokine signaling. Conversely, gut microbiota-derived metabolites and immune cells can also affect lung health. Research has shown that SCFAs produced by gut microbiota can modulate lung immune responses and influence the development of respiratory diseases (109). However, 16S rRNA profiling is cost-efficient but rarely resolves beyond genus level and cannot predict functional genes; shotgun metagenomics delivers species/strain identification and metabolic pathway data yet requires higher DNA input and bioinformatics load, while both methods yield compositional data that may bias cross-sample comparison of low-abundance taxa (110, 111). Furthermore, the composition and function of the lung microbiota in different lung cancer subtypes and disease stages are not well characterized (112). Zheng et al. (113) revealed distinct lung microbiota profiles in patients with NSCLC compared to healthy individuals, but the functional implications of these differences remain to be elucidated.

Chronic obstructive pulmonary disease is a common comorbidity in lung cancer patients and can significantly influence Gut-microbiota-lung Axis interactions (114). COPD is characterized by chronic inflammation and airflow limitation, and it is associated with alterations in both the gut and lung microbiota (21). However, the impact of COPD on microbiota-immune interactions in the context of lung cancer is not fully understood. Some studies suggest that COPD-related inflammation may exacerbate gut barrier dysfunction and promote the translocation of gut microbial products to the lungs, further intensifying immune responses (23, 114). For example, Bowerman et al. (30) found that patients with COPD had increased gut permeability and altered gut microbiota composition, which were associated with enhanced systemic inflammation. This inflammation could potentially influence lung cancer progression and treatment outcomes. Additionally, the shared risk factors and pathophysiological mechanisms between COPD and lung cancer may also affect microbiota-immune interactions (31, 115). However, more research is needed to clarify these complex relationships and to develop targeted interventions for lung cancer patients with COPD and other comorbidities.

Furthermore, translation of probiotics, FMT, or dietary modulation into thoracic oncology practice faces pragmatic barriers identified by Georgiou 2021 and updated trials. First, regulatory agencies lack harmonized criteria for live-biotherapeutic potency, leading to variable CFU counts between batches of *Clostridium butyricum* CBM588 (67). Second, FMT sourced from ICI-responders requires donor re-screening every 30 days to exclude transmissible pathogens, raising cost to ≈ US $3,500 per infusion in a recent US phase-I NSCLC protocol (NCT05122546), a figure incompatible with universal reimbursement. Third, dietary interventions such as 20 g day ^1^ resistant starch increased fecal butyrate by 2.3-fold in chemo-immunotherapy patients, yet adherence at 12 weeks was 54%, predominantly limited by grade 1–2 bloating (52). Fourth, antibiotic stewardship programs report that 38% of lung cancer admissions receive at least one course of broad-spectrum agents during treatment, potentially abrogating any microbiota-directed benefit; integration of rapid point-of-care pathogen identification could reduce unnecessary prescriptions, but prospective data in oncology are lacking. Collectively, these data indicate that microbiota-based adjuvants are feasible only within clinical trials or specialized centers equipped with GMP-grade biobanks and dietetic support; routine deployment outside such frameworks is currently premature.

## 9 Conclusion

In conclusion, the Gut-microbiota-lung Axis plays a crucial role in lung cancer development and treatment. Gut microbiota dysbiosis can impact lung health through immune, neural, and humoral pathways, and influence the efficacy of lung cancer therapies. Targeting the Gut-microbiota-lung Axis offers potential for enhancing treatment efficacy and improving patient outcomes. However, challenges such as the lack of standardized protocols and the need for personalized therapies remain. Further research is needed to fully elucidate the mechanisms underlying the Gut-microbiota-lung Axis in lung cancer and to translate these findings into clinical applications.
